# Corticospinal tract structure and descending motor output following anterior cruciate ligament reconstruction: an exploratory multimodal neuroimaging assessment

**DOI:** 10.3389/fnhum.2026.1888579

**Published:** 2026-09-08

**Authors:** HoWon Kim, Cole Geschwender, Adam S. Lepley

**Affiliations:** Performance, Rehabilitation, and Injury Management Through Exercise (PRIME) Lab, School of Kinesiology, University of Michigan, Ann Arbor, MI, United States

**Keywords:** active motor threshold, anterior cruciate ligament reconstruction, diffusion tensor image (DTI), functional magnet resonance imaging (fMRI), motor evoked potential (MEP), multimodal imaging, quadriceps muscle strength, transcranial magnetic stimulation (TMS)

## Abstract

**Background:**

Functional and structural differences in the central nervous system, including higher motor cortex activation, lower corticospinal excitability, and smaller corticospinal tract volume have been proposed as potential contributors to persistent quadriceps strength deficits following anterior cruciate ligament reconstruction (ACLR). However, prior work has largely examined these differences using isolated single modality neuroimaging approaches, limiting integration across brain activation, corticospinal excitability, and neural structure. Therefore, our primary aim was to compare neurophysiological and neurostructural outcomes between individuals with ACLR and uninjured individuals (CON) and to explore whether corticospinal tract (CST) volume conditioned the association between (1) cortical activation and corticospinal excitability and (2) corticospinal excitability and quadriceps strength.

**Methods:**

Nineteen right-foot dominant individuals, including 10 with a history of unilateral ACLR and 9 CON participated in the current study. Brain activation in primary motor cortex while performing a supine knee flexion-extension task, CST volume, and corticospinal excitability was measured by functional magnetic resonance imaging, diffusion tensor imaging, and transcranial magnetic stimulation, respectively. Group comparison and correlation analyses were conducted to evaluate the difference in quadriceps strength, brain activation, CST volume, and corticospinal excitability between the two groups and to measure association between these variables for each group, respectively. Exploratory moderation analyses were conducted to evaluate association between motor cortex activation, corticospinal excitability and MVIC, while determining whether this association is moderated by the structural volume of the CST.

**Results:**

Compared with controls, ACLR participants demonstrated higher active motor threshold, lower motor evoked potential (MEP) amplitude, and lower CST volume. CST volume was positively associated with MEP amplitude in the ACLR group. Exploratory moderation analyses yielded nominal interaction effects suggesting that CST volume may condition the relationships between task-related primary motor cortex activation and MEP amplitude, and between MEP amplitude and quadriceps strength in the ACLR group. Comparable interaction effects were not statistically detectable in controls.

**Conclusion:**

Individuals with ACLR demonstrated neurophysiological and neurostructural differences despite comparable quadriceps strength. Exploratory findings raise the hypothesis that CST volume may influence cross-modal relationships among brain activation, corticospinal excitability, and quadriceps strength after ACLR, highlighting the potential value of integrating structural and functional measures when evaluating the descending motor system. These findings provide a foundation for larger longitudinal investigations examining how CST structure interacts with neurophysiological function and motor recovery following ACLR.

## 1 Introduction

Arthrogenic muscle inhibition (AMI) has been discussed for more than four decades as a neurophysiological contributor to quadriceps dysfunction after knee trauma and as a potential therapeutic target (Stokes and Young, 1984). Yet persistent quadriceps weakness remains common after anterior cruciate ligament reconstruction (ACLR) despite standard rehabilitation (Brown et al., 2021), even among professional athletes with access to extensive rehabilitation resources (Herrington et al., 2021). Persistent quadriceps weakness is clinically important because it is associated with worse perceived function, altered landing biomechanics, increased risk of secondary ACL injury, and early onset of post-traumatic knee osteoarthritis (ØIestad et al., 2010). Therefore, improved understanding of ACLR-related neurophysiological alterations in motor control is needed to more effectively address persistent quadriceps weakness and improve functional outcomes.

Neuroimaging and neurophysiological studies suggest that ACLR is associated with changes across multiple levels of the descending motor system. Functional magnetic resonance imaging (fMRI) studies report greater task-related primary motor cortex (M1) activation (indirectly measured by blood-oxygen level dependent signal) during lower-extremity motor tasks in individuals after ACLR compared with matched controls (Grooms et al., 2017), yet M1 activation alone does not relate to quadriceps strength symmetry (Criss et al., 2023). In addition, transcranial magnetic stimulation (TMS) studies demonstrate increased threshold of corticospinal excitability of quadriceps muscle after ACLR (Rush et al., 2021; de Brito Santana and Baptista, 2025), although associations between threshold of corticospinal excitability and strength outcomes are inconsistent across studies (Héroux and Tremblay, 2006; Bodkin et al., 2019; Pietrosimone et al., 2013; Lepley et al., 2014; Scheurer et al., 2020), Collectively, these findings suggest that persistent quadriceps deficits after ACLR may reflect interacting alterations across cortical activation and corticospinal excitability that are not captured by any single neurophysiological measure.

Structural integrity of the descending motor pathway may further constrain these relationships. A diffusion tensor imaging (DTI) study has reported smaller corticospinal tract (CST) volume in the hemisphere contralateral to the ACLR limb compared to uninjured limb and positive associations between CST volume and corticospinal excitability on the ACLR limb (Lepley et al., 2019b). This finding highlights the potential importance of CST integrity as a moderator linking cortical activation to corticospinal output and, ultimately, muscle function. However, prior investigations have largely evaluated these factors in isolation, limiting understanding of how cortical activation, corticospinal excitability, and CST structure interact within the same individuals after ACLR and how these combined characteristics relate to quadriceps strength. Integrating multimodal neurophysiological assessments within a single cohort may provide a more comprehensive understanding of the neural determinants of persistent quadriceps weakness after ACLR.

Therefore, the purpose of this investigation was to employ fMRI, TMS, and DTI to evaluate neurophysiological characteristics influencing descending motor output following ACLR compared with matched uninjured controls. We aimed to explore the relationship between M1 activation and corticospinal excitability and determine whether this association is moderated by CST volume. Further, we examined whether corticospinal excitability is associated with quadriceps strength and this relationship is moderated by CST volume. We hypothesized that CST volume would significantly moderate both relationships.

## 2 Methods

A cross-sectional case–control study design was used to assess motor cortex activation, corticospinal excitability, and corticospinal tract structure during a single testing session. Data reported in this manuscript represent a subsample of participants included in previously published studies (Lepley et al., 2019a,b). A prior publication (Lepley et al., 2019a) evaluated neural and morphological differences between individuals with ACLR and uninjured controls. A subsequent publication (Lepley et al., 2019b) examined hemispheric differences in CST volume in individuals with a history of ACLR using a subsample from aforementioned cohort. The current manuscript addresses a distinct research question by examining whether CST volume moderates the relationships among M1 activation, corticospinal excitability, and quadriceps strength after ACLR. These moderation analyses were not reported in the prior publications. Data were collected at the University of Connecticut while the senior author was affiliated with that institution, under the original University of Connecticut ethics approval and funding (IRB number H16-178UCHC).

### 2.1 Participants

Ten individuals between the ages of 16–35 with a history of primary unilateral ACLR who were medically cleared to return to activity were recruited to participate. Additionally, ten healthy controls with no history of major lower extremity injury, matched by sex, age, height, weight, limb dominance, and activity level (Tegner Activity Scale) were also included. Individuals in both the ACLR and control groups were excluded if they reported any lower extremity injury in the past 6 months, history of a concussion/head injury in the past 6 months, previous loss of consciousness, history of a stroke, cranial neurosurgery, migraines, cancer in the brain, a diagnosed psychiatric or neurological disorder, current medications that alter neural activity, or imbedded intracranial metallic clips. The University’s Institutional Review Board approved study procedures and written informed consent was provided.

After data quality check, one participant in the control group was removed due to high noise in MRI data, thus 19 subjects were included in the current analysis.

### 2.2 MRI data

#### 2.2.1 Data acquisition

MRI data were acquired using a 3-Tesla Siemens Prisma scanner equipped with a 20-channel head/neck receive-only coil. Participants were positioned supine on the scanner table. To minimize head motion, Velcro straps were applied across the chest and pelvis, and foam pads were placed between the participant’s head and the head coil. Participants wore MRI-safe earphones to protect their hearing from scanner noise, to communicate with the research team, and to receive auditory cues during the motor task.

All participants underwent the same scanning protocol and acquisition order. Four types of brain scans were acquired in the following sequence: a high-resolution three-dimensional T1-weighted structural image, resting-state fMRI, task-based fMRI during a motor task, and diffusion tensor imaging (DTI).

##### 2.2.1.1 Structural image

A three-dimensional T1-weighted structural image was acquired using a magnetization-prepared rapid gradient-echo (MPRAGE) sequence with the following parameters: repetition time (TR) = 2,300 ms, echo time (TE) = 2.26 ms, inversion time (TI) = 900 ms, flip angle = 7°, field of view = 220 × 220 mm^2^, an acquisition matrix of 224 × 224, slice thickness = 1 mm, and 176 slices. Images were acquired with anterior–posterior in-plane phase encoding and isotropic voxel resolution of approximately 1 × 1 × 1 mm^3^. Slice oversampling (18.2%) was applied to reduce slab boundary artifacts.

##### 2.2.1.2 Task-based fMRI

Task-based fMRI was acquired using echo-planar imaging (EPI) sequence with the following parameters: TR = 3,000 ms, TE = 30 ms, flip angle = 80°, field of view = 200 × 200 mm^2^, an acquisition matrix = 100 × 100, slice thickness = 2.5 mm, and 60 slices. Images were acquired with anterior–posterior phase encoding with interleaved lice timing and anisotropic 2 × 2 × 2.5 mm^3^. During task-based fMRI sessions, a knee bolster was placed under each participant’s knees to maintain approximately 45° of knee flexion.

A unilateral knee flexion-extension task was used to investigate primary motor cortex (M1) activation during a simple, knee-focused lower extremity motor task (Grooms et al., 2015; Kapreli et al., 2006). Participants performed the task four times in total, completing two repetitions with each limb. A standardized order was applied for all participants: the non-dominant (left) limb was always tested first, followed by the dominant (right) limb, regardless of the injured side in participants with ACLR.

The unilateral knee flexion-extension task was performed as a 4.5-min block design consisting of nine 30-s blocks: four movement blocks interleaved with five rest blocks. Participants performed the task only during the movement blocks. The start and end of each movement block were cued auditorily. During movement blocks, a metronome sound at 1.27 Hz was played to pace the knee flexion-extension movement. Participants were instructed to extend the knee without fully locking it, and to flex it in time with the metronome, maintaining a consistent movement speed throughout the blocks.

##### 2.2.1.3 Diffusion tensor imaging

DTI was acquired using a single-shot EPI sequence with the following parameters: TR = 5,000 ms, TE = 69 ms, field of view = 256 × 256 mm2, acquisition matrix = 128 × 128, slice thickness = 3.0 mm, and 40 slices. Images were acquired with opposite phase encoding (anterior–posterior and posterior–anterior) using interleaved slice acquisition and anisotropic voxel size of 2.0 × 2.0 × 3.0 mm^3^. Diffusion weighting was applied along 36 non-collinear directions with a b-value of 1,000 s/mm^2^, along with one non-diffusion-weighted (b = 0) image.

#### 2.2.2 Data processing

##### 2.2.2.1 Task-based fMRI

fMRI data were preprocessed using FSL (Functional MRI of the Brain Software Library, version 6.0.5.2; FMRIB, Oxford, UK). Standard preprocessing steps included non-brain tissue removal (BET), slice timing correction (interleaved), intensity normalization, motion correction (MCFLIRT), and spatial smoothing with a 5 mm full-width at half-maximum (FWHM) kernel (Jenkinson et al., 2002; Smith et al., 2004). Functional images were linearly registered to each participant’s T1-weighted structural image (full search with boundary-based registration)and nonlinearly registered to the Montreal Neurological Institute (MNI) 2 mm template (full search with 12 degree of freedoms and 10 mm warp resolution) (Greve and Fischl, 2009). To minimize head motion artifacts, Independent Component Analysis-based Automatic Removal of Motion Artifacts (ICA-AROMA) was implemented (Pruim et al., 2015; Anand et al., 2020). After ICA-AROMA, a high-pass temporal filter (cut-off = 90 s) was applied to eliminate slow signal drift.

To segment each subject’s T1-weighted structural image into gray matter, white matter, and cerebrospinal fluid compartments, we used FAST (FMRIB’s Automated Segmentation Tool). Resulting white matter and cerebrospinal fluid masks were used to create subject-specific regressors, which were included as nuisance covariates in the subject-level analysis to regress out signals arising from these tissues, thereby prioritizing gray matter activity (Pruim et al., 2015; Kim et al., 2025; Zuleger et al., 2024).

Subject-level general linear model (GLM) analyses were performed to identify brain regions showing increased activation during movement blocks compared to rest blocks. These analyses were conducted separately for each of the four unilateral knee flexion-extension task conditions (two tasks per limb). For each subject, second-level fixed-effect GLMs were used to calculate average activation across repeated tasks for each limb. Group-level analyses employed third-level mixed-effects modeling using FLAME 1 + 2, with individual gray matter volume included as a nuisance covariate to control for differences in gray matter volume. Statistical significance for all fMRI analyses was determined using cluster correction for multiple comparisons (threshold: z > 3.1, *p* < 0.05).

Group-level task activation maps for each limb were used to define regions of interest (ROIs). Task activation maps and contralateral primary motor cortex (M1) masks from the Harvard-Oxford Cortical Atlas were binarized using FSL’s fslmaths. These binarized maps were added together, and the overlapping region (identified by an intensity value of 2) defined limb-specific, functionally and anatomically based ROIs. For each subject, individualized ROIs were generated by locating the peak voxel (highest z-score) within these limb-specific ROIs using FSL’s fslstats, then creating a 6 mm spherical ROI around this coordinate using fslmaths. Mean percent signal change within each individualized ROI was extracted with FSL’s featquery, averaged per limb. For subsequent analyses, contralateral M1 activation corresponding to the injured limb in the ACLR group and the non-dominant limb in the control group was used.

##### 2.2.2.2 Diffusion tensor imaging

T1-weighted structural images were aligned to echo phase encoding directions (anterior–posterior and posterior–anterior) for improved registration, then reconstructed using the FreeSurfer software suite (version 6.0; http://surfer.nmr.mgh.harvard.edu, Laboratory for Computational Neuroimaging, Martinos Center for Biomedical Imaging, Boston, MA, USA) with standard parameters. Morphometric segmentation of gray matter, white matter, and cerebrospinal fluid was performed using FreeSurfer’s automated pipeline.

DTI data were preprocessed using TORTOISE (version 3.1.1) for correction of motion, eddy current distortions, and EPI geometric distortions. The corticospinal tract (CST) was reconstructed for each participant using TRACULA, which generates probabilistic tractography based on native-space diffusion images. For each participant, CST volume was measured in the hemisphere contralateral to the injured limb for the ACLR group and contralateral to the non-dominant limb for the control group. CST volume was then normalized to total intracranial volume (TIV) to account for inter-individual differences in brain size. Normalized CST volume was used in subsequent analyses.

### 2.3 Corticospinal excitability via transcranial magnetic stimulation

Motor evoked potentials (MEP) elicited during TMS were used to quantify corticospinal excitability for the vastus medialis muscle of the injured limb. To obtain the electromyography (EMG) signal during a TMS data acquisition, the distal vastus medialis muscle belly was prepped with two 10 mm, pre-gelled Ag–AgCl (EL503, BIOPAC Systems Inc., Goleta, CA, USA) disc-shaped surface EMG electrodes. AcqKnowledge 4.0 software (BIOPAC Systems Inc.) was used to visualize EMG signals. EMG signals were amplified with a gain of 1,000 (EMG100C BIOPAC Systems Inc.) before being digitally converted with a 16-bit data acquisition system (MP150 BIOPAC Systems Inc.). EMG signal was collected at 2 kHz with a common mode rejection ratio of 110 dB, a noise voltage of 0.2 μV and an input impedance of 1 M.

TMS testing took place in a standard testing chair (MagVenture Treatment Chair, 9016B008 MagVenture Inc., Atlanta, GA) with participants’ knees and hips at 90° of flexion and wearing a Lycra swim cap with 0.5 cm grid to identify an optimal stimulating point. Prior to TMS testing, a total of three maximal voluntary isometric knee extension contractions (MVIC) were assessed via a belt stabilized handheld dynamometer (microFET; Hoggann Scientific LLC, West Jordan, UT, USA), and the average of the three trials were used as a target MVIC. During TMS testing, participants produced voluntary isometric quadriceps contractions of 10% of their MVIC, which was objectively assessed and monitored via the belt stabilized handheld dynamometer with visual feedback. MVIC data was normalized by participant body weight and also used in subsequent analyses. One control participant did not complete the MVIC test; therefore, MVIC data were available for eight control participants.

A double cone angled TMS coil (D-B80, MagVenture Inc., Atlanta, GA, USA) was used for stimulation. A trained research member moved the coil in increments of 0.5 cm in anterior-to posterior and medial-to-lateral directions until the optimal stimulating point was detected, defined as the location producing the greatest MEP amplitude at 60% of stimulator output. Active motor thresholds (AMT) were established by detecting the lowest TMS intensity required to evoke a measurable (> 100 μV) MEP in 5/10 trials at the stimulation point. Five MEPs were elicited at 120% of AMT. Muscle responses of vastus medialis were elicited using peripheral electrical nerve stimulation on femoral nerve immediately after MEP measures. The average of five peak-to-peak MEP amplitudes were normalized to the average of three maximal muscle responses. Normalized MEP values from the injured limb in the ACLR group and the non-dominant limb in the control group were used in subsequent analyses.

### 2.4 Statistical analysis

All statistical analyses were conducted in SPSS Statistics (version 31.0; IBM, Armonk, NY, USA), with a significance threshold of 0.05. Prior to group comparisons, assumptions of normality and homogeneity of variance were assessed using the Shapiro–Wilk and Levene’s tests, respectively. When assumptions were met, group differences between the ACLR and control groups in primary variables, including M1 activation, MEP, CST volume, MVIC, and AMT, were analyzed using Welch’s t-tests based on the unequal sample size. Where the assumption of normality was violated (MEP), Mann–Whitney U tests were used. For all analyses, data from the injured limb in the ACLR group and the non-dominant limb in the control group were used.

Hedges’ *g* effect sizes were calculated for each group comparison and interpreted as small (*g* ≈ 0.2), medium (*g* ≈ 0.5), or large (*g* ≈ 0.8). Correlational analyses were performed separately for each group to determine the relationship between outcome variables. Pearson correlation coefficients were used for association analyses between variables when data distributions were normal; otherwise, Spearman’s rho was applied (MEP).

Moderation analyses utilized the PROCESS macro (version 5.0) for SPSS to evaluate whether CST volume moderated the relationships between M1 activation and MEP, and between MEP and MVIC, respectively. Each group underwent separate moderation analyses using Model 1 in PROCESS and a 100,000-permutation resampling approach, with interaction terms included in both models. Permutation resampling was used to support inference but does not address overfitting or leverage sensitivity. Each model included three predictors plus the intercept, resulting in four estimated parameters and six residual degrees of freedom in each group. Adjusted R^2^ was reported in addition to R^2^. Influence diagnostics were evaluated using Cook’s distance, with values greater than 4/n considered potentially influential. Leave-one-out analyses were performed to examine whether interaction terms were sensitive to removal of individual participants. All continuous predictors and moderators were mean centered prior to regression analyses. Regression assumptions were checked, including normality (Shapiro–Wilk test), linearity (visual inspection of residual plots), homoscedasticity (studentized Breusch-Pagan test), and multicollinearity (variance inflation factors [VIF < 5]). To provide further context for the moderation analysis, Johnson-Neyman analyses were applied to determine CST volume values where the effect of the predictor on the outcome became statistically significant (*p* < 0.05). Simple slopes plots were generated to visualize conditional effects of the moderator at the mean and at ±1 standard deviation.

Given the small sample size, moderation analyses were considered exploratory and preliminary in nature, with analyses intended to characterize potential relationships among neurophysiological, neurostructural, and strength-related outcomes. Accordingly, group comparisons, correlation analyses, and moderation analyses were treated as exploratory, and no Bonferroni or false discovery rate (FDR) corrections were applied. Reported *p*-values are unadjusted and should be interpreted as nominal and hypothesis-generating.

## 3 Results

There was no difference in demographics between groups. The ACLR group displayed lower MEP and CST volume and higher AMT compared with the control group (*p* < 0.05). There was no difference in M1 activation (*p* = 0.07) and MVIC (*p* = 0.80) between groups (Table 1).

**Table 1:** Demographics and dependent variables.

Variables	ACLR (*n* = 10)	CON (*n* = 9)	*p* values	Hedges’ g
Age (years)	22.60 ± 1.96	23.11 ± 1.76	0.56	0.26
Height (cm)	166.37 ± 7.5	165.95 ± 9.59	0.92	0.05
Weight (kg)	65.41 ± 12.6	62.60 ± 10.52	0.60	0.23
Sex (M:F)	6:4	6:3	–	–
Tegner physical activity level	7.20 ± 1.45	8.78 ± 1.99	0.07	0.91
Months from surgery	70 ± 23.62	–	–	–
Injured side (L:R)	3:7	–	–	–
M1 activation	1.20 ± 0.87	0.62 ± 0.27	0.07	0.89
MEP*	0.014 ± 0.008	0.037 ± 0.023	0.02	1.27
CST volume*	0.0032 ± 0.0006	0.0037 ± 0.0004	0.04	1.04
AMT*	49.4 ± 10.09	37.11 ± 5.56	0.005	1.42
MVIC (Nm/kg)^a^	2.97 ± 0.58	3.06 ± 0.83	0.80	0.12

Data is presented as mean ± standard deviation. ^*^Statistically significant group difference at *p* < 0.05.

a There were only 8 participants in the control group due to missing data. ACLR, anterior cruciate ligament reconstruction; CON, control; M1 activation, primary motor cortex activation; MEP, motor evoked potentials; CST volume, corticospinal tract volume; AMT, active motor threshold; MVIC, quadriceps maximal voluntary isometric contraction.

**Figure 1:**
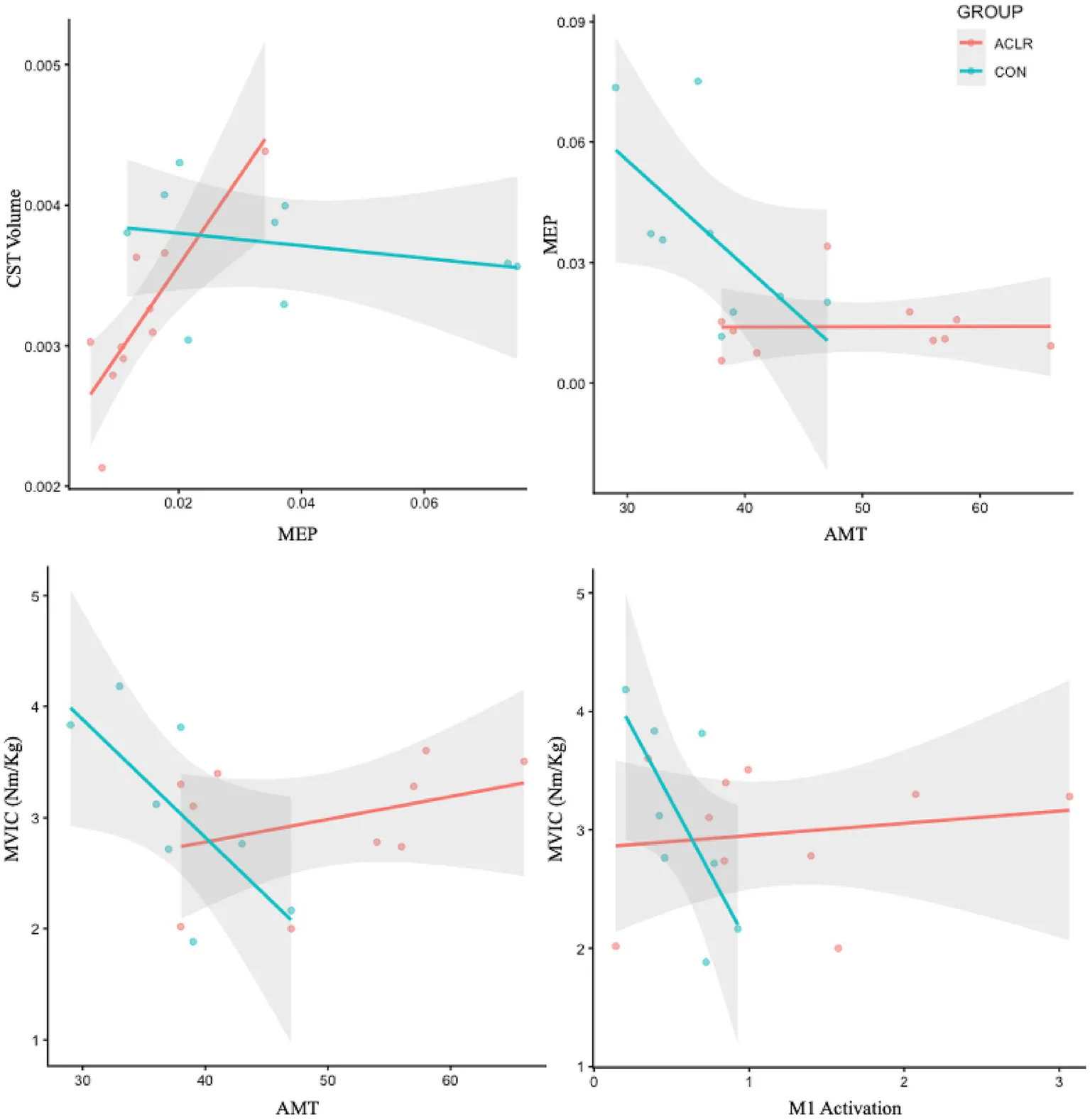
Each group exhibited distinct associations among primary motor cortex (M1) activation, motor evoke potential (MEP), corticospinal tract (CST) volume, quadriceps maximum voluntary isometric contraction (MVIC), and active motor threshold (AMT). In the anterior cruciate ligament reconstruction (ACLR) group, CST volume was positively associated with MEP. In the control (CON) group, MVIC was negatively associated with M1 activation and AMT.

MEP and CST volume were positively correlated in the ACLR group (*r* = 0.82, *p* = 0.004). In the control group, three negative associations approached the nominal significance threshold. AMT was negatively associated with MVIC (*r* = −0.71, *p* = 0.047) and MEP (*ρ* = −0.67, *p* = 0.050), and M1 activation was negatively associated with MVIC (*r* = −0.71, *p* = 0.048). These findings were unadjusted for multiple comparisons and should be interpreted cautiously given the small sample size and number of correlations examined. There were no other significant correlations among other variables (Table 2; Figure 1).

**Table 2:** Correlation matrix of neurophysiological, structural, and strength variables by group.

	Control					
	Variables	M1 Activation	AMT	MEP^+^	CST	MVIC
ACLR	M1 activation	–	0.35 (0.35)	−0.28 (0.46)	0.25 (0.52)	−0.71 (0.048)^#^
	AMT	0.14 (0.70)	–	−0.67 (0.05)^#^	0.3 (0.44)	−0.71 (0.047)^#^
	MEP^+^	0.39 (0.26)	0.12 (0.75)	–	−0.38 (0.31)	0.36 (0.39)
	CST	0.14 (0.70)	−0.11 (0.75)	0.82 (0.004)^*^	–	−0.31 (0.45)
	MVIC	0.15 (0.67)	0.36 (0.31)	−0.13 (0.73)	−0.56 (0.09)	–

Values are correlation coefficients with *p*-values in parentheses. The upper triangle represents the control group, and the lower triangle represents the ACLR group. Pearson’s r was used for correlations among normally distributed variables; Spearman’s ρ was used for correlations involving MEP due to its non-normal distribution. *p*-values are unadjusted for multiple comparisons and should be interpreted as nominal and exploratory. *p < 0.05. ACLR *n* = 10; control *n* = 10, except analyses involving MVIC in controls included *n* = 9 due to one missing MVIC test. ACLR, anterior cruciate ligament reconstruction; M1 activation, primary motor cortex activation; AMT, active motor threshold; MEP, motor evoked potential; CST, corticospinal tract volume; MVIC, maximal voluntary isometric contraction.

### 3.1 Moderation analysis of M1 activation, CST, and MEP

**Figure 2:**
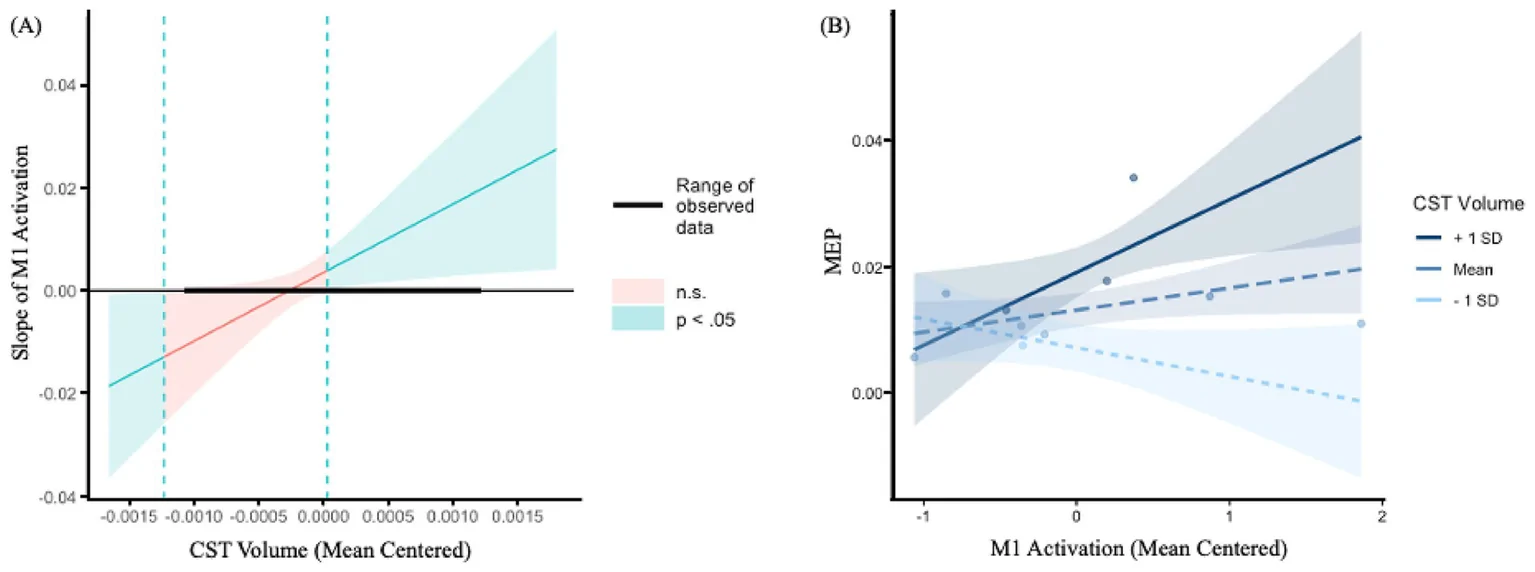
Moderation analysis in the anterior cruciate ligament reconstruction (ACLR) group showing that corticospinal tract (CST) volume moderates the association between primary motor cortex (M1) activation and motor evoked potential (MEP). Axes display mean-centered values. **(A)** Johnson–Neyman analysis identified CST values > 0.00003 (mean-centered [raw value of 0.00323]) as the region in which the effect of M1 activation on MEP was statistically significant (*p* < 0.05); below this value, the effect was not significant. **(B)** Simple slopes (conditional effects) at low (−1 *SD*), mean, and high (+1 *SD*) CST volume indicate that M1 activation was positively associated with MEP at high CST volume (*b* = 0.0121, *p* = 0.025), but not at mean or low CST volume (*p* = 0.137 and *p* = 0.215, respectively). CST volume was normalized to TIV and retained as a proportion; therefore, large unstandardized coefficients reflect the small numerical scale of normalized CST volume. Shaded bands represent 95% confidence intervals, which are wide due to the small sample size and uncertainty of the model estimates.

In ACLR group, moderation analysis revealed that CST volume significantly moderated the relationship between M1 activation and MEP. The regression model was significant, explaining 89% of the variance in MEP (*R*^2^ = 0.89, adjusted *R*^2^ = 0.83, *F* (3, 6) = 15.47, *p* = 0.003). The interaction term (M1 activation × CST volume) was statistically significant and explained 15% more variance in MEP (Δ*R*^2^ = 0.15, *b* = 13.32, *p* = 0.032), indicating that CST volume moderates the relationship between M1 activation and MEP (Figure 2). All statistical assumptions were met.

Cook’s distance identified two potentially influential observations exceeding the threshold of 0.40, ACLR 1 (Cook’s D = 0.61) and ACLR 5 (Cook’s D = 1.35). Sensitivity analyses excluding these observations showed that the interaction retained the same direction and the overall interpretation remained unchanged and statistically significant. Leave-one-out analyses showed that the interaction retained the same direction across all 10 iterations, although statistical significance was not retained in 3 iterations. These results suggest a directionally consistent but statistically fragile moderation effect.

In control group, there was no moderation effects of CST on the relationship between M1 activation and MEP. The regression model explaining 22% of the variance in MEP (*R*^2^ = 0.22, *F* (3, 5) = 0.38, *p* = 0.77) and the interaction term was not statistically significant (*b* = −32.86, *p* = 0.76).

### 3.2 Moderation analysis of MEP, CST, and MVIC

In ACLR group, moderation analysis revealed that CST volume also significantly moderated the effect of MEP on MVIC. The regression model was significant, explaining 77% of variance in MVIC (*R*^2^ = 0.77, adjusted *R*^2^ = 0.66, *F* (3, 6) = 6.72, *p* = 0.024). The interaction term (MEP × CST volume) was statistically significant and explained 42% more variance in MVIC (Δ*R*^2^ = 0.42, *b* = −91,498, *p* = 0.016), indicating that CST volume significantly moderated the relationship between MEP and MVIC. The negative coefficient for the interaction suggests that as CST increases, the effect of MEP on MVIC decreases. All the assumptions were satisfied, except for high multicollinearity involving MEP (VIF = 8.44).

Cook’s distance identified two potentially influential observations exceeding the threshold of 0.44: ACLR 5 (Cook’s D = 0.99) and ACLR 6 (Cook’s D = 0.48). Sensitivity analyses indicated that the interaction retained the same direction after removal of influential observations, but statistical significance was not consistently retained, when Subject 5 was removed. Leave-one-out analyses showed the same pattern: the interaction direction was retained across all 10 iterations, but significance was lost in 2 iterations. These results suggest a directionally consistent but statistically fragile moderation effect.

In control group, there was no moderation effects of CST on the relationship between MEP and MVIC. The regression model explaining 23% of the variance in MEP (*R*^2^ = 0.23, *F* (3, 5) = 0.51, *p* = 0.69) and the interaction term was not statistically significant (*b* = 56,925, *p* = 0.66).

## 4 Discussion

The overall aim of this study was to evaluate neurophysiological and neurostructural factors related to the descending motor system following ACLR and to examine the relationship between motor cortex activation, corticospinal excitability and MVIC, while determining whether this association is moderated by the structural volume of the CST. Consistent with prior researches sharing sample (Lepley et al., 2019a,b), group comparisons demonstrated that ACLR participants exhibited lower CST volume, reduced MEP, and increased AMT relative to controls. In addition, the positive association between CST volume and MEP amplitude observed in the ACLR group provides support for a link between structure of CST volume and function of corticospinal excitability (Lepley et al., 2019b).

Overall, individuals after ACLR demonstrated neurophysiological and neurostructural differences despite having quadriceps MVIC comparable to controls. These findings suggest that recovery of maximal force output alone may not reflect complete restoration of central motor system function, particularly after clearance to return to physical activity. Exploratory moderation analyses indicated that CST volume may condition relationships among M1 activation, MEP amplitude, and quadriceps strength.

### 4.1 Neurophysiological characteristics in ACLR group

The combination of higher AMT and lower MEP amplitude in ACLR participants is consistent with a neuro-inefficiency pattern in which greater central recruitment may be required to achieve a given motor output (Grooms et al., 2017; Schnittjer et al., 2023). Neuro-inefficiency can be conceptualized as the counterpart to the neural efficiency hypothesis, in which skilled performers often show less resource-intensive neural activation than novices during comparable tasks (Li and Smith, 2021; Naito and Hirose, 2014; Yang, 2015). In this content, higher AMT reflects increased threshold for corticospinal excitability, indicating that greater magnetic stimulation intensity via TMS, or greater physiological activation, is required to elicit an action potential to the target muscle. Lower MEP amplitude indicates reduced corticospinal excitability, which can reflect combined supraspinal and spinal contributions to the responsiveness of the corticomotor pathway (Chen et al., 2008; Chen, 2000).

Although M1 activation during the knee motor task was not statistically different between groups, the large effect size in the ACLR group may reflect greater task-related cortical engagement and associated metabolic cost. Also, the observed difference in M1 activation was greater than minimum detectable change for a lower extremity motor task (Kim et al., 2025) and was consistent in direction with prior findings (Grooms et al., 2017). Spinal-reflexive excitability has been reported to normalize, and in some cases increase above baseline levels, in the chronic phase after ACLR (> 24 months) (Rush et al., 2021; Rodriguez et al., 2021). Thus, for participants in the current study who were at least 31 months post-ACLR, the observed differences in AMT, MEP, and M1 activation may reflect long-term supraspinal and corticospinal adaptations rather than ongoing spinal-level impairment. Collectively, these findings support the interpretation that, even long after ACLR and return to activity, central motor system inefficiencies may persist and influence the generation of motor output.

Beyond functional measures, we observed structural differences in CST volume between the ACLR and controls. Prior reports of lower fractional anisotropy and higher mean diffusivity after ACLR suggest that CST microstructure, not only tract volume, may be altered (Lepley et al., 2019b; Yu et al., 2025). Because CST structure is critical for efficient descending motor signal and ascending sensory signal propagation (Lemon and Griffiths, 2005), reductions in volume and microstructure are plausibly associated with slower conduction, temporal dispersion, and less effective motor unit recruitment (Betti et al., 2022). Consistent with this framework, CST volume was positively correlated with MEP amplitude in the ACLR group, supporting a relationship in which reduced CST integrity may constrain corticospinal output.

In control group, the greater CST volume observed may reduce the extent to which pathway structure constrains corticospinal excitability, which could explain why the CST volume–MEP association observed after ACLR was not statistically detectable in controls, suggesting that CST structure may become more relevant to corticospinal excitability following ACLR. This interpretation remains preliminary because the associations were not formally compared between groups. Moreover, comparable MVIC does not necessarily indicate complete neuromuscular recovery, as maximal strength may not capture compensatory motor strategies or subtle alterations in neural control. Prospective studies are needed to determine whether these alterations are associated with secondary ACL injury.

### 4.2 Moderation effects of CST

Exploratory moderation analyses in the ACLR group suggested that the association between task-related M1 activation and MEP amplitude may vary with CST volume. Greater M1 activation was associated with larger MEP amplitudes at relatively greater CST volumes within the observed sample, whereas this association was not statistically detectable at lower CST volumes (Figure 2). This pattern generates the hypothesis that CST structure may constrain the extent to which task-related cortical engagement corresponds to corticospinal excitability after ACLR. The CST is the principal conduit for descending motor commands (Lemon and Griffiths, 2005), and structural characteristics of the tract can influence the organization and propagation of descending motor signals (Betti et al., 2022). Accordingly, relatively greater CST volume may support stronger coupling between cortical activation and corticospinal excitability, whereas lower CST volume may represent a structural constraint that weakens this association. This may propose “bottleneck” mechanism.

**Figure 3:**
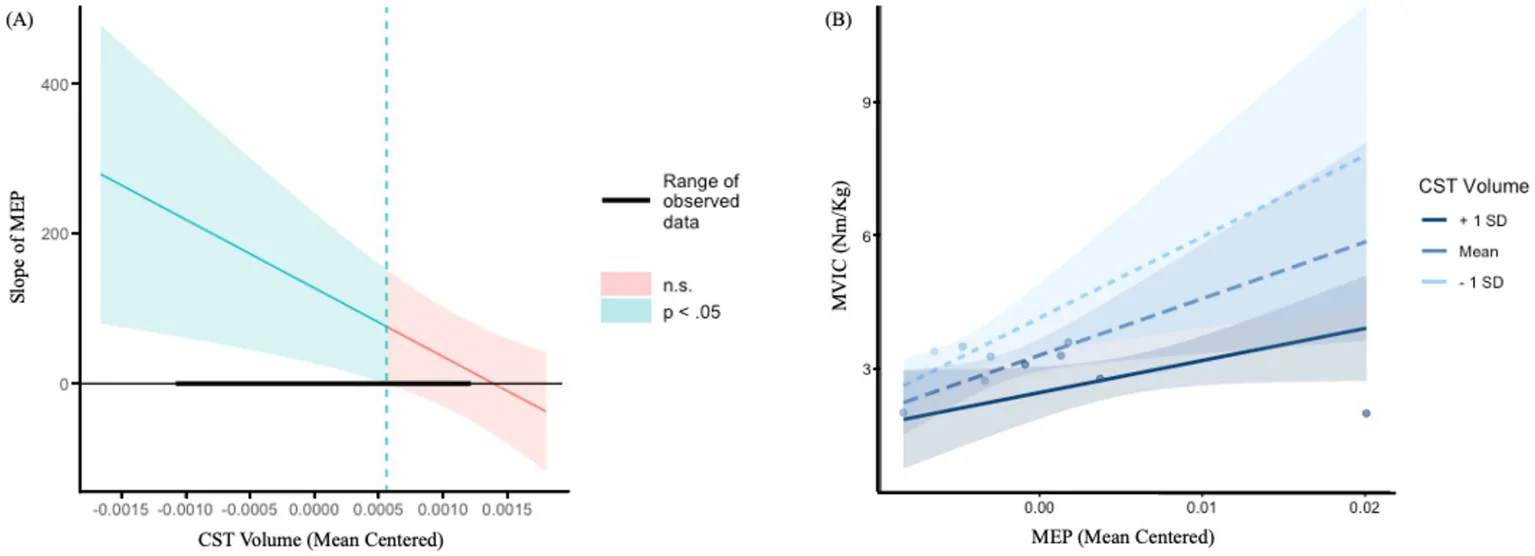
Moderation analysis in the Anterior Cruciate Ligament Reconstruction (ACLR) group showing that Corticospinal Tract (CST) volume moderates the association between Motor Evoked Potential (MEP) and Quadriceps Maximal Voluntary Isometric Contraction (MVIC). Axes display mean-centered values. **(A)** Johnson–Neyman analysis identified CST values < 0.00056 (mean-centered [raw value of 0.00375]) as the region in which the effect of MEP on MVIC was statistically significant (*p* < 0.05); above this value, the effect was not significant. **(B)** Simple slopes (conditional effects) at low (−1 SD), mean, and high (+1 SD) CST volume indicate that MEP was positively associated with MVIC at low CST (*b* = 178.22, *p* = 0.016) and at mean CST (*b* = 138.98, *p* = 0.019), but not at high CST (*p* = 0.063). CST volume was normalized to TIV and retained as a proportion; therefore, large unstandardized coefficients reflect the small numerical scale of normalized CST volume. Shaded bands represent 95% confidence intervals, which are wide due to the small sample size and uncertainty of the model estimates.

A different conditional pattern observed for the negative relationship between MEP amplitude and MVIC. Their association was strongest at lower CST volumes and diminished as CST volume increased (Figure 3) one possible interpretation is that, at lower CST volumes, quadriceps strength is more closely associated with corticospinal excitability because the functional capacity of the corticospinal pathway may place a greater constraint on force production. At greater CST volumes, maximal strength may be less dependent on variation in corticospinal excitability and more strongly influenced by peripheral and task-related contributors, including muscle morphology, fiber characteristics, motor-unit behavior, voluntary activation, and intermuscular coordination. This interpretation could explain why the MEP–MVIC association weakened as CST volume increased. However, because this model exhibited substantial multicollinearity involving MEP and sensitivity to individual observations, the observed pattern is statistically fragile and should be regarded as a hypothesis for future testing rather than evidence of an established moderation mechanism. Nonetheless, the pattern suggests that future studies should examine whether CST structure influences how descending motor system function relates to quadriceps force production after ACLR (Héroux and Tremblay, 2006; Bodkin et al., 2019; Pietrosimone et al., 2013).

Collectively, these exploratory patterns raise the hypothesis that CST structure may influence neuromuscular function at more than one level after ACLR. Relatively greater CST volume may support stronger coupling between task-related cortical activation and corticospinal excitability, whereas lower CST volume may increase the apparent dependence of quadriceps strength on corticospinal excitability. These apparently opposing conditional effects are not necessarily contradictory, rather they may reflect different constraints along the pathway from cortical engagement to corticospinal excitability and, ultimately, maximal force production. Comparable MVIC between groups further suggests that recovery of maximal strength may coexist with altered neural strategies, although the present data cannot determine whether such patterns represent successful compensation or persistent neural inefficiency.

This hypothesis is consistent with a broader motor recovery principle observed in neurological populations, where the structural integrity of descending motor pathways is related to motor function and recovery potential (Stinear et al., 2007; Lindenberg et al., 2010; Byblow et al., 2015). ACLR differs fundamentally from stroke because it does not involve a direct CNS lesion, and the present study did not assess interhemispheric reorganization or functional connectivity. Nevertheless, both bodies of work support the more general premise that motor output reflects interactions between structural pathway capacity and functional neural recruitment. In ACLR, these interactions could arise from long-term neurostructural remodeling (Terada et al., 2019; Pijnenburg et al., 2014) related to altered joint afference, changes in limb use and physical activity, pain-related adaptations, or persistent compensatory motor behavior. White matter pathways exhibit activity-dependent plasticity, but CST volume alone cannot distinguish pre-existing variation from adaptive remodeling, maladaptive plasticity, or progressive structural change. Longitudinal studies combining tract volume, diffusion-based microstructural measures, neurophysiology, and motor outcomes are needed to test this integrated model.

### 4.3 Limitation

Several limitations should be considered when interpreting these findings. First, the small sample size limits statistical power, precision, and generalizability, particularly for the group-specific moderation analyses. Each moderation model included three predictors and an intercept, leaving only six residual degrees of freedom in the ACLR group and increasing the risk of overfitting, unstable interaction estimates, and sensitivity to individual observations. Although influence and leave-one-out analyses indicated that the interaction coefficients retained the same direction and were not solely attributable to one participant, statistical significance was not retained in every iteration, indicating that the findings were statistically fragile. The MEP × CST model predicting MVIC warrants additional caution because of substantial multicollinearity involving MEP (VIF = 8.44), which limits the stability and independent interpretation of its coefficients. Moreover, because moderation models were estimated separately by group, an interaction observed in the ACLR group but not statistically detectable in controls does not establish a between-group difference in moderation. Multiple exploratory group comparisons, correlations, and moderation analyses were also conducted without correction for multiple testing, increasing the possibility of Type I error, while the small sample increased the risk of Type II error for nonsignificant findings. Permutation resampling provides an alternative basis for statistical inference but does not address overfitting, leverage, or model instability. Accordingly, all moderation findings should be considered exploratory and hypothesis-generating.

Second, the cross-sectional design precludes causal inference and does not establish the temporal ordering of neurophysiological and neurostructural differences after ACLR. Although all ACLR participants were in the chronic postoperative phase and had been cleared to return to physical activity, time since surgery varied considerably. In addition, the control group reported higher Tegner activity levels than the ACLR group, with a large between-group effect size. Habitual physical activity may influence CST structure, corticospinal excitability, and neuromuscular performance and therefore represents a potential confound. Exploratory analyses did not identify statistically significant associations between time since surgery or Tegner activity level and the primary outcomes. However, these analyses were underpowered and cannot exclude their influence. Larger longitudinal studies incorporating objective wearable-derived activity measures are needed to characterize how neural outcomes evolve after ACLR and whether they vary by postoperative time, activity exposure, sex, graft type, or concomitant injury.

Third, although multimodal measurements were collected from the same cohort, the fMRI, TMS, and strength assessments were performed under different motor conditions. M1 activation was assessed using the task-related blood-oxygen-level-dependent response during dynamic, paced knee flexion and extension, whereas MEPs were elicited from the vastus medialis during a static contraction at 10% MVIC. MVIC, in contrast, represented maximal force production by the quadriceps muscle group. These measures therefore differ in contraction mode, force demand, joint motion, proprioceptive feedback, and motor-unit recruitment requirements. Furthermore, the M1 activation response does not directly quantify efferent motor output (Hillman, 2014; Kim and Ogawa, 2012), and MEP amplitude reflects combined supraspinal, spinal, peripheral, and methodological influences (Spampinato et al., 2023). Relationships among M1 activation, MEP amplitude, and MVIC should consequently be interpreted as cross-modal associations among complementary aspects of the descending motor system rather than direct physiological coupling under identical task demands. The muscle-specific MEP measurement also may not fully represent the neural control of whole-quadriceps force production.

Finally, MEPs were elicited at 120% of each participant’s AMT, a standard method for normalizing suprathreshold stimulation to individual motor threshold. Because AMT was higher in the ACLR group, the absolute stimulation intensity used to elicit MEPs was also higher. However, lower MEP amplitude observed despite this higher intensity is unlikely to result from insufficient stimulation, but MEPs obtained at a single threshold-relative intensity cannot distinguish differences in motor threshold from differences in corticospinal recruitment gain. In addition, CST volume is a macroscopic measure and cannot determine whether observed structural differences reflect axonal density, myelination, fiber organization, or other microstructural processes. Future studies should incorporate more closely matched tasks across modalities, concurrent force and EMG measurements, full TMS stimulus–response curves, advanced diffusion measures, and larger longitudinal samples to test the preliminary structure–function relationships identified here.

## 5 Conclusion

In summary, the integrated use of fMRI, TMS, and DTI provided preliminary evidence that CST volume may be an important structural factor related to neurophysiological function after ACLR. Individuals with ACLR demonstrated altered corticospinal and cortical characteristics, and exploratory moderation analyses suggested that CST volume may condition relationships among task-related M1 activation, corticospinal excitability, and quadriceps strength. These findings should be interpreted as hypothesis-generating given the small sample size, cross-sectional design, and cross-modal differences in task demands across fMRI, TMS, and strength assessments. Nevertheless, the results highlight the potential value of multimodal neurophysiological assessment for understanding persistent neuromuscular alterations after ACLR. Future longitudinal studies with larger samples are needed to confirm these relationships, determine the temporal evolution of CST structure and function, and evaluate whether neurophysiologically informed rehabilitation strategies can improve motor recovery after ACLR.

## Data Availability

The raw data supporting the conclusions of this article will be made available by the authors, without undue reservation.
